# Structural alterations of skeletal muscle in copd

**DOI:** 10.3389/fphys.2014.00104

**Published:** 2014-03-19

**Authors:** Sunita Mathur, Dina Brooks, Celso R. F. Carvalho

**Affiliations:** ^1^Department of Physical Therapy, University of TorontoToronto, ON, Canada; ^2^Department of Physical Therapy, University of São PauloSão Paulo, Brazil

**Keywords:** COPD, skeletal muscle, deconditioning, muscle fiber types, mytochondria, protein balance

## Abstract

**Background:** Chronic obstructive pulmonary disease (COPD) is a respiratory disease associated with a systemic inflammatory response. Peripheral muscle dysfunction has been well characterized in individuals with COPD and results from a complex interaction between systemic and local factors.

**Objective**: In this narrative review, we will describe muscle wasting in people with COPD, the associated structural changes, muscle regenerative capacity and possible mechanisms for muscle wasting. We will also discuss how structural changes relate to impaired muscle function and mobility in people with COPD.

**Key Observations**: Approximately 30–40% of individuals with COPD experience muscle mass depletion. Furthermore, muscle atrophy is a predictor of physical function and mortality in this population. Associated structural changes include a decreased proportion and size of type-I fibers, reduced oxidative capacity and mitochondrial density mainly in the quadriceps. Observations related to impaired muscle regenerative capacity in individuals with COPD include a lower proportion of central nuclei in the presence or absence of muscle atrophy and decreased maximal telomere length, which has been correlated with reduced muscle cross-sectional area. Potential mechanisms for muscle wasting in COPD may include excessive production of reactive oxygen species (ROS), altered amino acid metabolism and lower expression of peroxisome proliferator-activated receptors-gamma-coactivator 1-alpha mRNA. Despite a moderate relationship between muscle atrophy and function, impairments in oxidative metabolism only seems weakly related to muscle function.

**Conclusion**: This review article demonstrates the cellular modifications in the peripheral muscle of people with COPD and describes the evidence of its relationship to muscle function. Future research will focus on rehabilitation strategies to improve muscle wasting and maximize function.

## Introduction

Chronic obstructive pulmonary disease (COPD) is the fourth leading cause of death worldwide and presents a major burden of disease in middle and high-income countries (World Health Organization, 2008). COPD is primarily a disease of the respiratory system and is diagnosed based on abnormal lung function evaluated by spirometry (low forced expiratory volume); and symptoms such as dyspnea, chronic cough and/or sputum production (Vestbo et al., 2013). However, impairments in lung function and breathing only explain one aspect of the disability experienced by individuals with COPD. There are several secondary consequences of COPD and skeletal muscle dysfunction poses a key limitation in these patients. In fact, muscle mass has been shown to be an independent predictor of mortality from lung function in people with COPD (Marquis et al., 2002; Schols et al., 2005). Muscle size and muscle strength are also associated with important clinical outcomes such as reduced quality of life (Mostert et al., 2000), greater healthcare resource utilization (Decramer et al., 1996) and exercise intolerance in this population (Hamilton et al., 1995; Gosselink et al., 1996).

The concept that skeletal muscle dysfunction could be an important limitation to exercise capacity in people with COPD was first described by Killian et al. (1992) in a study where the symptoms limiting peak exercise capacity were systematically assessed using ratings of perceived exertion. Approximately 40% of people with COPD had an early termination of exercise due to symptoms of leg fatigue which were greater than their rating of shortness of breath at the end of a progressive exercise test; and in another 30% of patients, ratings of leg fatigue and dyspnea were equal. These findings led to further investigations of skeletal muscle dysfunction in people with COPD including examinations of muscle fiber typing (Jobin et al., 1998; Whittom et al., 1998), mitochondrial enzyme concentrations (Maltais et al., 2000), muscle metabolism (Kutsuzawa et al., 1995), functional deficits in muscle strength (Gosselink et al., 2000) and endurance (Serres et al., 1998) and the relationship between muscle function and peak exercise capacity (Hamilton et al., 1995; Gosselink et al., 1996). The early literature on skeletal muscle dysfunction in COPD was summarized in a comprehensive review by the American Thoracic Society and European Respiratory Society (ATS/ERS, 1999). The body of literature on skeletal muscle dysfunction has continued to explode and recent studies suggest that about one third of patients with COPD exhibit quadriceps muscle weakness (Seymour et al., 2010) and that muscle weakness and muscle atrophy are observed not only in moderate to severe disease but also earlier in the course of the disease (Seymour et al., 2010; Shrikrishna et al., 2012; Kelly et al., 2013).

Several factors are thought to contribute to the development of skeletal muscle dysfunction in people with COPD. Low physical inactivity or the effects of chronic deconditioning is likely a major factor contributing to muscle dysfunction in patients with COPD, who tend to be sedentary (Pitta et al., 2005; Watz et al., 2009). Although a causal link between inactivity and muscle dysfunction is difficult to establish in COPD, low physical activity has been associated with quadriceps muscle wasting even in people with mild airflow obstruction (Shrikrishna et al., 2012). Typically, muscle disuse atrophy has a pattern of inducing greater lower limb atrophy and weakness compared to upper limb (de Boer et al., 2008; Pisot et al., 2008). This pattern has also been observed in people with COPD where hadgrip strength tends to be better preserved than lower limb strength (Gosselink et al., 2000; Heijdra et al., 2003). Furthermore, studies examining muscle strength during and after an acute exacerbation of COPD, demonstrate that muscle weakness is apparent as early as the third day of the exacerbation (Spruit et al., 2003) and further decreased by about 5% after 5 days of hospitalization. The result of this study also showed that handgrip force also declined during the hospitalization initially, but did not decline further with longer hospitalization as it did for quadriceps force (Spruit et al., 2003). These finding suggests that muscle disuse is an important factor contributing to weakness in people with COPD.

Other factors that are associated with skeletal muscle dysfunction include the use of oral corticosteroid medications that causes “steroid-induced myopathy” in COPD (Decramer et al., 1996); low circulation testosterone (Van Vliet et al., 2005); hypoxemia (Koechlin et al., 2005), nutritional depletion (Engelen et al., 1994), oxidative stress (Couillard et al., 2003) and systemic inflammation (Spruit et al., 2003). Exposure to tobacco smoke, the main cause of COPD, is also recognized to contribute to muscle dysfunction even prior to the development of lung disease. In an observational study of over a 1000 healthy young adults (21–36 years old), an inverse relationship between smoking tobacco and knee extensor muscle strength was reported (Kok et al., 2012). Furthermore, this study demonstrated that 100 g of tobacco a week resulted in a reduction of 3% in muscle strength in men and 5% in women and this association existed independently of lifestyle physical fitness and body fat percentage (Kok et al., 2012). In addition, an inverse relationship between smoking tobacco and increased fatigability (Wüst et al., 2008) and muscle fiber atrophy (Montes de Oca et al., 2008) has also been described in non-COPD smokers. These results clearly demonstrate that smoking has an adverse effect of muscle function even in healthy subjects without COPD diagnosis. The combination of these factors likely leads to the complex set of adaptations that is observed in the peripheral muscles of people with COPD.

The purpose of this narrative review is to describe structural changes in skeletal muscle of people with COPD and describe the main mechanisms hypothesized to contribute to muscle atrophy. The relationship between skeletal muscle dysfunction to physical function and mobility will also be discussed.

## Structural changes in the skeletal muscle

### Skeletal muscle atrophy in COPD

Approximately 30–40% of people with COPD experience muscle atrophy (Schols et al., 1993; Engelen et al., 1994; Vermeeren et al., 2006) and a proportion of these patients may present normal body mass since the amount of fat mass is relatively maintained (Engelen et al., 1999; Eid et al., 2001; Vermeeren et al., 2006). Loss of muscle mass has been observed at the whole body level using D-XA and bioelectrical impedance measures, at the level of individual muscles using computed tomography (Bernard et al., 1998), magnetic resonance imaging (Mathur et al., 2007, 2008) and ultrasound (Seymour et al., 2010), as well as from muscle biopsy studies (Whittom et al., 1998; Gosker et al., 2002a,b). In an early study by Marquis et al. (2002), it was found that mid-thigh cross-sectional area measured using CT was a stronger predictor of mortality than lung function (FEV_1_) in a large cohort of patients with moderate to severe COPD. Similarly Schols et al. (2005) found that fat-free mass but not fat mass was an independent predictor of survival.

Muscle atrophy has important consequences for mobility, as muscle strength and power are closely related with muscle size. Furthermore, the loss of muscle mass and quality have been shown to have important multi-system consequences in other chronic disease conditions. For example in diabetes, muscle atrophy and fat infiltration is associated with poor glucose tolerance (Goodpaster et al., 2003) and may also be related to the immunologic status of an individual (Jo et al., 2012). These multi-system issues require further attention in the COPD population. The mechanism of muscle atrophy and how it may be accelerated in people with COPD is a growing area of investigation.

### Muscle fiber type

The first study evaluating peripheral muscle fiber types in people with COPD was performed by Hughes et al. (1983) using biopsy analyses from the quadriceps of patients with moderate COPD. While they did not observe any change in fiber type proportions, they observed a significant atrophy in the type II fibers that was associated with weight loss. Since then, several studies have quantified changes in proportion and size in skeletal muscle fiber types of the lower limb of COPD and they have mainly focused in the vastus lateralis (VL) muscle. Jakobsson et al. (1990) showed a reduced percentage of type I fibers in the quadriceps, which was confirmed by the findings of Whittom et al. (1998). Another study in patients with COPD reported fiber-type analysis by quantifying myosin heavy-chain (MHC) and myosin light-chain (MLC) isoforms and observed a significantly greater proportion of MHC-2B in the VL compared with control subjects (Satta et al., 1997). They also observed that the pattern of distribution of MLC isoforms was shifted toward fast isoforms in COPD patients. A meta-analysis established a pathological proportion of slow to fast fiber types in people with COPD patients aged 60–70 years old. The authors evaluated eight studies with 84 patients and determined that, compared with reference values, a proportion of type I fibers less than 27% and of type IIX fibers greater than 29% in the VL could be defined as pathological (Gosker et al., 2007b). Gosker et al. suggested that COPD patients present a reduction in the type I fibers that is strongly associated with the severity of the disease.

Although the precise causes for such changes is not fully understood, it has been suggested that multiple factors such as hypoxemia and long-term disuse are related to the higher proportion of type II fibers. Hildebrand et al. (1991) reported that the high proportion of type II fibers was positively correlated with hypoxemia suggesting this may be a factor underlying muscle fiber differentiation in COPD (Hildebrand et al., 1991). It also appears that these changes are more profound in the lower limb muscles since no changes have been observed in the proportion of type I fibers of the biceps brachii muscle of people with severe COPD and matched control subjects (Sato et al., 1997); which suggests that disuse may play a role in fiber type changes in COPD.

### Metabolic enzymes

There is substantial evidence to suggest a decrease in the activity of key oxidative enzymes in peripheral muscles of COPD patients, such as citrate synthase and succinate dehydrogenase. Muscle biopsy from the VL muscle in people with severe COPD have demonstrated lower oxidative enzyme activity compared with healthy subjects; however, no significant difference was observed in the activity of the glycolytic enzyme between COPD and controls (Jakobsson et al., 1995; Maltais et al., 1996). Other studies have found increased activity of glycolytic enzymes such as phosphofructokinase (Whittom et al., 1998). It has been also suggested that hypoxemia contributes to these metabolic alterations in people with COPD; however, no reversal in the activities of any enzyme was observed even after long-term oxygen therapy (Jakobsson et al., 1995). Interestingly, muscle cytochrome oxidase activity is inversely related to arterial oxygen levels (PaO_2_) in COPD patients what suggests a compensatory response to reduced O_2_ availability to augment ATP production (Wagner, 2006).

### Muscle capillarity

The oxidative metabolism in skeletal muscle is dependent on mitochondrial volume, density and activity and on muscle blood supply, therefore alterations in the muscle capillary network or mitochondria can lead to decreased exercise tolerance in COPD. A study by Simard et al. (1996) reported that the number of capillaries per surface area in the VL of patients with COPD was 53% lower than in age-matched normal subjects. Similarly, Jobin et al. (1998) observed a lower number of capillaries per square millimeter and lower ratio of capillaries per fiber ratio in COPD patients compared with controls. However, when normalized for fiber cross-sectional area, the number of capillary contacts per fiber were similar between patients with COPD and control subjects. A possible explanation for the reduced number of capillaries could be hypoxemia; however, patients from Jobin's study did not present with marked hypoxemia either at rest or during exercise. Only recently, Eliason et al. (2010) provided evidence of a disturbed muscle-to-capillary interface in COPD patients and a positive correlation between the degree of muscle capillarization, airflow obstruction and exercise capacity. The authors also showed that muscle capillarization decreased with the severity of the disease. Hypoxemia may be a possible explanation for the reduced vascularization. This hypothesis is strengthened by findings that COPD patients present an overexpression of the von Hippel-Lindau tumor suppression protein (Jatta et al., 2009) that lead to an adverse effect on tissue capillarization and impair the transduction of hypoxic-angiogenetic transcription factors such as vascular endothelial growth factor (Kondo and Kaelin, 2001).

### Mitochondria dysfunction

Although cardiac output and ventilatory limitation have been observed during exercise in COPD patients (Cuttica et al., 2011), there is a noteworthy observation that the oxidative capacity of peripheral skeletal muscle is significant and remains reduced even following lung transplantation (Lands et al., 1999). Several studies have suggested that mitochondrial dysfunction exists in people with COPD. At least three changes in mitochondria have been suggested: an impairment in density and biogenesis, increased oxidative stress and apoptosis (Meyer et al., 2013). Gosker et al. (2007a) reported a reduction in the mitochondrial area along with reduced proportion of type I fibers suggesting that the reduction in the number of mitochondria might be related to changes slow type fibers. The role of oxidative stress in mitochondrial dysfunction was observed by Puente-Maestu et al. (2012) and recently reviewed by Kirkham and Barnes (2013). The highest sources of reactive oxygen species (ROS) are the alveolar macrophages and activated neutrophils from the circulation that release superoxide radicals and hydrogen peroxide. ROS are chemically reactive molecules containing oxygen, and include oxygen ions and peroxides. These are natural byproducts of normal oxygen metabolism and have important roles in cell signaling and homeostasis. In addition, mitochondrial respiration can also generate ROS due to the constant exposure to sources of inflammatory responses to bacterial and viral infections within the lungs. For instance, airway epithelial cells exposed to lipid soluble components from the tobacco induce the production of mitochondria-derived ROS (van der Toorn et al., 2009). Mitochondrial dysfunction has also been described in the airway epithelium of chronic cigarette smokers. Hoffmann and coworkers evaluated changes in mitochondrial morphology and expression of markers for mitochondrial capacity in the human bronchial epithelial cell line from ex-smokers with COPD (GOLD stage IV) and compared with age-matched smoking and never-smoking controls. Their results demonstrated that long-term cigarette smoking induces robust and persistent changes in mitochondrial structure and function in human bronchial epithelial cells, including increased fragmentation, branching, density of the matrix and reduced numbers of cristae. Interestingly, they also showed that most of these changes persisted upon smoking cessation. The authors speculated that an attenuated antioxidant response with elevated ROS in COPD may lead to an increased oxidant burden, possibly contributing to the observed mitochondrial defects in bronchial epithelial cells from COPD patients (Hoffmann et al., 2013). Hara and colleagues compared mitochondrial morphology in lung tissues from smokers without COPD and from COPD patients (Hara et al., 2013). Analyzing the electron microscopic of lung tissues, they demonstrated that mitochondria in bronchial epithelial cells tended to be fragmented in COPD but not in smokers without COPD, suggesting the fission process dominancy of mitochondrial dynamics in COPD pathogenesis. Tobacco smoke can also affect mitochondrial function in skeletal muscle. There is evidence that carbon monoxide has a direct inhibitory effect *in vitro* on cytochrome *c* oxidase (an enzyme related to the ATP synthesis in the mitochondria) activity in the human VL (Alonso et al., 2003); as well as its classical effect of oxygen depletion (Young and Caughey, 1990). Tobacco smoke may also act on muscle mitochondria through an increased expression of tumor necrosis factor-α (Tang et al., 2010).

## Mechanisms of muscle atrophy in COPD

There are several interactive mechanisms that may contribute to the underlying development of muscle wasting in people with COPD. For a thorough review of the pathways leading to muscle atrophy, the reader to referred to the review by Langen et al. (2013). We present a brief overview of the major mechanisms that have been studied to date in the section below.

### Regulation of myonuclear turnover

Muscle apoptosis was hypothesized to be one factor underlying the development of muscle atrophy in COPD, however there are limited data in this area. Agusti et al. (2002) found that TUNEL positive nuclei were higher in people with COPD who had a low BMI; and there was an inverse relationship between TUNEL and BMI. However, specific measures of muscle size were not included in this study. Barreiro et al. (2011) found a relationship between muscle mass and apoptotic nuclei in people with severe COPD but no difference in caspase-3 between the COPD patients and healthy controls. Lastly, Gosker et al. (2003) found no evidence of active caspase-3 in muscle fibers of people with COPD and TUNEL-positive fibers were similar between people with COPD and healthy controls. However, the authors did report changes in the muscle fibers indicative of impaired muscle regenerative capacity such as the presence of fibrosis and adipocyte replacement in the muscle tissue.

There are some data to suggest that muscle regenerative capacity may be impaired in people with COPD. Theriault et al. (2012) described significantly shorter telomere lengths in people with COPD with low mid-thigh cross-sectional area. Also, people with COPD who had relatively preserved muscle mass had a significantly higher proportion of central nuclei, indicating past muscle regeneration. Although these results were based on a limited sample of 16 patients with COPD, they provide some evidence for exhausted muscle regenerative capacity in people with COPD who present with low muscle mass. Other markers of muscle regeneration such as Myf5, MyoD and myogenin have been shown to be similar between COPD patients and controls (Plant et al., 2010); whereas other studies have found a difference in these factors between COPD patients and controls; and between cachetic and non-cachetic patients with COPD (Vogiatzis et al., 2010; Fermoselle et al., 2012). Myostatin, an inhibitor of muscle growth, has been shown to be higher in COPD patients compared with controls (Man et al., 2010; Plant et al., 2010; Ju and Chen, 2012).

### Regulation of protein balance

Muscle mass is regulated through a balance of protein synthesis and degradation. In COPD, it is not clear whether protein synthesis is downregulated, protein degradation is upregulated or whether muscle mass depletion is a result of both processes. One of the proteolytic systems which has been studied in COPD is the ubiquitin-protease system. There is evidence for increased activation of this system in people with COPD, which may contribute to muscle wasting (Fermoselle et al., 2012; Lemire et al., 2012). People with COPD who exhibit muscle atrophy also have increased levels of atrogin-1 and MuRF-1 (muscle ring finger 1), both of which regulators of muscle atrophy (Plant et al., 2010; Lemire et al., 2012) as well as regulators FOXO-1 and FOXO-3 (Doucet et al., 2007; Debigare et al., 2010). The autophagy-lysosome pathway has also been hypothesized to be a factor in protein degrading leading to muscle wasting in people with COPD (Hussain and Sandri, 2013). A recent study found evidence for autophagy in the VL and tibialis anterior muscles of people with COPD (Guo et al., 2013). The number of autophagosomes was also inversely correlated with FEV_1_; and the degree of lipidation of the LCB3 protein was associated with low thigh muscle cross-sectional area. Further studies are needed to understand the contributions of the ubiquitin-proteasome pathway and autophagy-lysosome pathway in muscle wasting in COPD.

In terms of protein synthesis, the IGF-1-Akt pathway has been studied in people with COPD, however the results are conflicting. Circulating levels of IGF-1 have been shown to be similar between people with COPD and controls across disease severities (Piehl-Aulin et al., 2009) and in cachetic vs. non-cachetic patients with COPD (Debigare et al., 2003). However, during periods of acute exacerbation, IGF-1 levels have been shown to be decreased (Crul et al., 2007). The discrepancy in findings may be due to heterogeneous patient samples and stability of the disease; periods of acute exacerbation for example, are known to result in muscle atrophy.

## Clinical relevance of muscle atrophy in COPD

Reduced exercise capacity, poor quality of life, difficulty with activities of daily living and recurrent acute exacerbation are not simply the consequence of pulmonary impairment, but also impacted by peripheral muscle dysfunction.

Although a causal relationship has not been established, significant association have been observed between measures of muscle function, lung function and exercise performance. Lower quadriceps strength is correlated with lower FEV_1_ (Bernard et al., 1998). In addition, lower extremity muscle strength is significantly correlated with the 6-min walking distance, incremental shuttle walk performance, maximal oxygen update and symptoms on incremental exercise test but not endurance shuttle walking test performance (Hamilton et al., 1995; Gosselink et al., 1996; Saey et al., 2003; Steiner et al., 2005). More specifically, reduced fat free mass is correlated with decreased walking distance and maximal oxygen uptake (Schols et al., 1991; Baarends et al., 1997).

Reduced limb muscle strength also contributes to increased dyspnea, poor quality of life and health status (Shoup et al., 1997; Mostert et al., 2000); whereas improvement in muscle strength result in better in quality of life (Simpson et al., 1992). Furthermore, quadriceps wasting is independently associated with lower levels of physical activity in early COPD disease (Shrikrishna et al., 2012). In addition, there is a tendency for patients with muscle wasting to have higher depression scores than those without wasting (Chavannes et al., 2005; Al-shair et al., 2009) although the direction of the relationship is not known. On one hand, depression could result in altered appetite and decrease physical activity contributing to muscle wasting. On the other, muscle wasting could impact the ability to perform activities of daily living as well as community integration and therefore lead to depression and isolation.

Although lower extremity strength has received most of the attention, upper extremity strength is also compromised in individuals with COPD. The force-generating capacity of upper limb muscles are reduced in patients with COPD compared to healthy control (Gosselink et al., 2000). Specifically, arm elevation affects lung volume and respiratory muscles resulting in reduction in force-generating capacity (Janaudis-Ferreira et al., 2009). The reduction in upper extremity muscle strength in COPD contributes to difficulties in performing arm activities.

Finally, there is increasing evidence of impaired postural control in patients with COPD (Butcher et al., 2004; Beauchamp et al., 2009; Roig et al., 2009). Although the underlying mechanisms for reduced postural control among individuals with COPD remain unclear, many hypotheses have been proposed, including decreased levels of physical activity, peripheral muscle weakness and altered trunk muscle mechanics among others (Butcher et al., 2004; Beauchamp et al., 2009; Roig et al., 2009). Thus, skeletal muscle dysfunction may play an important role in the balance impairment in individuals with COPD.

Impaired exercise capacity and peripheral muscle dysfunction contribute to increased mortality and reduced health status, even when accounting for age and lung function status (Marquis et al., 2002; Schols et al., 2005). Specifically, mid-thigh muscle cross sectional area, an index of muscle mass has a strong impact on mortality in individuals with FEV1 of less than 50% (Schols et al., 2005). Although lower body mass index is a predictor of mortality in individuals with COPD, loss of muscle has more implications for survival than loss in other compartments (Schols et al., 2005). Decramer et al. (1997) also found that lower quadriceps muscle force was more strongly associated with higher utilization of health care resources compared to pulmonary function and exercise capacity.

## Conclusion

Despite COPD being primarily a respiratory disease, these individuals present with important secondary dysfunction in their peripheral muscles characterized by muscle atrophy alterations in muscle fiber type, fiber composition as well as reduction in oxidative enzymatic activity, capillarity and mitochondrial dysfunction. These muscle changes have important clinical consequences such as impaired exercise tolerance, low physical activity and quality of life in people with COPD.

### Conflict of interest statement

The authors declare that the research was conducted in the absence of any commercial or financial relationships that could be construed as a potential conflict of interest.
